# Exploring the effect of a microencapsulated citrus essential oil on *in vitro* fermentation kinetics of pig gut microbiota

**DOI:** 10.3389/fmicb.2022.952706

**Published:** 2022-08-29

**Authors:** Carmen M. S. Ambrosio, Izabella D. Alvim, Caifang Wen, Ruth Gómez Expósito, Steven Aalvink, Carmen J. Contreras Castillo, Eduardo M. Da Gloria, Hauke Smidt

**Affiliations:** ^1^Dirección de Investigación, Innovación y Responsabilidad Social, Universidad Privada del Norte (UPN), Trujillo, Peru; ^2^Laboratory of Microbiology, Wageningen University & Research, Wageningen, Netherlands; ^3^Technology Center of Cereal and Chocolate, Institute of Food Technology (ITAL), São Paulo, Brazil; ^4^Nestlé Institute of Health Sciences, Société des Produits Nestlé S. A., Lausanne, Switzerland; ^5^Department of Agri-Food Industry, Food and Nutrition, “Luiz de Queiroz” College of Agriculture (ESALQ), University of São Paulo, São Paulo, Brazil; ^6^Department of Biological Science, “Luiz de Queiroz” College of Agriculture (ESALQ), University of São Paulo, São Paulo, Brazil

**Keywords:** citrus essential oil, microencapsulation, pig gut microbiota, SCFA, antibiotics

## Abstract

Essential oils (EOs) have emerged as a potential alternative to antibiotics in pig breeding due to their antimicrobial properties. Citrus EOs, a common by-product of the orange juice industry, can be an interesting alternative from a financial perspective due to their huge offer in the global market. Thus, the effect of a citrus EO, and specifically different formulations of Brazilian Orange Terpenes (BOT), on pig gut microbiota was evaluated by means of an *in vitro* fermentation model simulating different sections of the pig gut (stomach, ileum, and colon). Treatments consisted in: BOT in its unprotected form (BOT, 1.85 and 3.70 mg/mL), microencapsulated BOT (MBOT, 3.50 and 7.00 mg/mL), colistin (2 μg/mL), and a control. BOT and MBOT altered in a similar way the total bacterial 16S rRNA gene copies in the stomach only from 18 h of incubation onwards, and no metabolite production in terms of short-chain fatty acids (SCFAs) was detected. In ileal and colonic fermentations, BOT and MBOT affected ileal and colonic microbiota in terms of total bacterial 16S rRNA gene copies, reduced phylogenetic diversity, and altered composition (*p* < 0.05) as evidenced by the significant reduction of certain bacterial taxa. However, more pronounced effects were found for MBOT, indicating its higher antimicrobial effects compared to the unprotected BOT, and suggesting that the antibacterial efficiency of the unprotected BOT was probably enhanced by microencapsulation. Furthermore, MBOT stimulated lactate production in ileal fermentations and greatly stimulated overall SCFA production in colonic fermentations. This indicates that besides the shifts in ileal and colonic microbiota by the delivered EO (BOT), the wall material of microcapsules (chitosan/modified starch) might have worked as an additional carbon source with prebiotic functioning, stimulating growth and metabolic activity (SCFAs) of colonic bacteria.

## Introduction

The interest of using phytogenic feed additives in animal breeding, such as essential oils (EOs), has been growing during the last 20 years. This is because antibiotic use as a feed additive to improve growth in farm animals was fully banned in 2006 by the European Union (EC 1334/2003, 2003), particularly due to the concern of its use being related to the increase of antibiotic resistance in human bacteria. Especially in pig production, antibiotics have contributed to improve the productivity of this sector by controlling enteric diseases in young pigs (Burch et al., 2008). EOs are plant extracts constituted by a mix of about 20–60 aromatic-volatile compounds (Burt, 2004; Bakkali et al., 2008). These compounds confer to EOs several biological properties, turning them into a potential alternative to antibiotics in animal feed such as in pig production. Specifically, due to their antimicrobial, anti-inflammatory, and antioxidant properties, EOs could exert beneficial effects on the pig gut ecosystem (Omonijo et al., 2018), which would impact positively on the pig performance. A preferred effect of an EO is the modulation of the gut microbiota of pigs by suppressing pathogenic bacteria without compromising beneficial commensal bacteria. Previous *in vitro* studies using pure cultures of pig gut bacteria have shown the efficiency of EOs or EO compounds in inhibiting pathogenic bacteria such as *Escherichia coli* and *Clostridium perfringens* without negatively affecting beneficial bacteria such as *Lactobacillus* sp. and *Bifidobacterium* sp. (Si et al., 2006; Ouwehand et al., 2010; Ambrosio et al., 2017). To this end, also EOs from citrus plants have been demonstrated to have this selective antimicrobial activity in an *in vitro* study (Ambrosio et al., 2017).

Often, citrus EOs are a vital by-product of the citrus processing industries. These oils are the most produced EOs around the world, with the majority coming from orange EOs (Iwabuchi et al., 2010; Barbieri and Borsotto, 2018). It has been estimated that the worldwide orange EO production reached 60.3 thousand tons in 2020, with Brazil being the major producing country, representing 57.7% of the global production (United Nations, 2021). Thus, due to the selective antimicrobial activity, the vast availability in the global market and the safety recognition to be used as a feed additive (Fisher and Phillips, 2008), the application of citrus EOs in pig feed could be feasible.

Nonetheless, an important aspect to consider is that the effectiveness of an EO on bacterial species may vary from pure cultures to that in mixed communities, as in the case of bacteria that are found in the pig gut environment. *In vitro* gut fermentation models are advantageous alternative assays that enable the stable cultivation of a complete intestinal microbiota allowing to investigate the effects of supplements on microbiota composition and functionality under defined experimental conditions (Payne et al., 2012). Due to practical and ethical reasons, costs, and the high reproducibility between experiments, *in vitro* gut fermentation models represent an excellent alternative to *in vivo* assays (Gresse et al., 2017). For instance, a previous study has reported the use of an *in vitro* fermentation set-up to simulate the gastric, jejunal, and cecal sections of the pig gut to test the effect of several EO compounds on microbiota composition and fermentation pattern of the gut of pigs (Michiels et al., 2009).

A few studies have proven that citrus peel EOs, alone or in combination with other EOs, can be potential natural growth promoters for broiler chickens, since positive effects on gut microbiota and microbial metabolites, as well as in gut morphology were found (Hong et al., 2012; Erhan et al., 2017). Similarly, in-feed citrus peel extracts have led to an enhanced growth performance (Ebrahimi et al., 2014) and proximal intestinal morphology of broiler chickens (Akbarian et al., 2013). Mostly, isolated EO compounds such as thymol, carvacrol and cinnamaldehyde have been widely proven for having a modulatory effect on pig gut microbiota in *in vivo* studies (Li S. Y. et al., 2012; Li et al., 2018). Furthermore, EOs or EO compounds can enhance the production of digestive secretions and nutrient absorption, reduce the pathogenic stress, exert antioxidant activity, and reinforce the pig immune system (Zeng et al., 2015). In addition, EOs/EO compounds may affect the organoleptic properties of the feedstuff, enhancing its flavor and palatability, which would impact positively on the feed intake, especially in pigs (Franz et al., 2010; van der Aar et al., 2017).

As outlined above, several studies have investigated the effect of EOs on animal performance, gut microbiota composition, and immune system. However, an important aspect that should be considered when EOs are intended to be added to the feedstuff is the volatile nature of the EOs, since when an EO is added in its pure form, loss of its effectiveness due to volatilization can occur during feedstuff storage. Moreover, it is necessary to consider an effective mode to deliver the EO to the pig gut. To this end, encapsulation techniques such as microencapsulation could assist to solve volatilization problems, preservation of EO properties, and delivery of EO to the pig gut (Omonijo et al., 2018). Therefore, this study aimed at investigating the effect of a microencapsulated commercial citrus EO, Brazilian Orange Terpenes (BOT), on pig gut microbiota by means of an *in vitro* fermentation model simulating several sections of the pig gut.

## Materials and methods

### Essential oil supply

Brazilian orange terpenes (BOT) was the commercial citrus EO used in this study, which was a by-product of orange juice production. It was supplied by a factory from São Paulo State, Brazil. Once the sample was received, it was kept refrigerated (4°C) in amber bottles until further use. Then, this oil was used as a core material for microencapsulation by spray-drying using a mixture of chitosan and modified starch as the wall material (Ambrosio et al., 2020). The amount of BOT oil used was 1:4 (w/w) relative to the mass of the wall material. The total BOT oil content in the microcapsules was 13.2% (Ambrosio et al., 2020). BOT oil was characterized for having a major compound limonene (78.65% in non-polar column, 79.38% in polar column) followed by minor compounds such as *cis*-limonene oxide, *trans*-limonene oxide, *trans*-carveol, carvone, *trans*-p-mentha-2,8-dien-1-ol, myrcene, *cis*-p-mentha-2,8-dien-1-ol, and *cis*-carveol among others as reported by Ambrosio et al. (2019, 2020).

### Preparation of EO/antibiotic solutions

Stock solutions of the unprotected BOT oil were prepared at 10.3 and 20.6% v/v (92.5 and 185 mg/mL, respectively) with sterile distilled water using Tween 80 (T09799RA, Exôdo científica-Brazil) as an emulsifier. Regarding the microencapsulated BOT (MBOT), stock solutions were prepared at 178.7 and 357 mg/mL with sterile distilled water. Colistin sulfate stock solution (C4461; Sigma-Aldrich) was prepared at 0.1 mg/mL with sterile distilled water.

### *In vitro* fermentation assay

The *in vitro* fermentation assay was carried out under simulated prevailing conditions of the stomach, ileum, and colon of pigs. Specific artificial media for each condition were used in combination with the unprotected BOT or MBOT, in order to evaluate the effect of the different EO preparations on the fermentation kinetics and metabolite production of pig gut microbiota.

#### Media preparation

For simulating the prevailing conditions of the stomach, ileum, and colon of pigs in the *in vitro* system, three different artificial media were prepared following the protocols reported in previous studies and as summarized in Supplementary Table S1. The stomach medium was prepared according to Beumer et al. (1992) with slight modifications. In brief, once the basal solution for the stomach simulation was prepared, it was boiled and cooled, and the pH of the medium was adjusted to 3 with HCl. Then, 47.5 mL of this basal solution was dispensed into 120 mL serum bottles that were sealed with butylrubber stoppers and aluminum crimp seals. Next, the bottles were autoclaved. Once the medium was cooled to room temperature, 2.5 mL of the enzyme solution (previously filter-sterilized, Supplementary Table S1) was added to the bottles using a syringe under sterile conditions. The ileum medium was prepared following the recipe by Blake et al. (2003) with slight modifications (Supplementary Table S1). In brief, once the ileum-basal solution was prepared; it was boiled, cooled by flushing with N_2_/CO_2_ (80:20 v/v), and the pH was adjusted to 6. Then, 50 mL of this basal solution was dispensed into serum bottles, which were sealed with butylrubber stoppers and aluminum caps. Next, bottles were flushed with N_2_/CO_2_ to remove O_2_, bottles were autoclaved and cooled. Afterward, filter-sterilized vitamin and trace element solutions were added to the bottles (0.05 and 0.10 mL, respectively), using a syringe and under sterile conditions. The colon medium was prepared according to the recipe by Williams et al. (2005) with slight modifications (Supplementary Table S1). In brief, the colon-basal solution was prepared, boiled, cooled by flushing with N_2_/CO_2_ (80:20 v/v) and the pH was adjusted (pH = 6.5–7). Afterward, 46.4 mL of this basal solution were distributed into serum bottles. Then, bottles were flushed with N_2_/CO_2_ and sealed with butylrubber stoppers and aluminum caps. Next, bottles were autoclaved and cooled, and 0.6 mL of vitamin/phosphate solution (filter-sterilized), 2.4 mL of bicarbonate solution, and 0.6 mL of reducing agent (Supplementary Table S1) were added to the bottles using a syringe under sterile conditions. Bottles with the corresponding media were kept at 4°C until use (2 days at most).

#### Inoculum preparation

Stomach, ileum, and colon contents were collected from commercial male pigs in a slaughterhouse located in the province of Gelderland, the Netherlands. Immediately after the pigs were slaughtered, contents from each pig's gut location were collected and immediately stored under anoxic conditions, placing the gut content in sterile serum bottles previously flushed with N_2_. After the bottles were sealed with a butylrubber stopper and aluminum crimp seals, a sterile needle coupled to a filter (0.2 μm) was inserted into the rubber stopper, and bottles were placed into an anaerobic jar containing anoxic gas generating sachets (Thermo Scientific™ Oxoid AnaeroGen). The collected samples were rapidly transferred to the laboratory, within 2 h at most. Once in the laboratory, the material from each gut location was weighted and samples from 3 pigs were pooled for each location inside of an anaerobic tent filled with a gas mixture of 96% N_2_/4% H_2_. Stomach, ileum, and colon inocula were separately prepared from each of the generated pools with a pre-warming step (39°C), anaerobic (N_2_/CO_2_) and sterile saline solution (0.9% v/v NaCl) in a ratio of 1:1, 1:10 and 1:10 (w/w), respectively. Inocula were prepared right before the experiment began to be used as fresh as possible.

#### *In vitro* fermentation procedure

The *in vitro* fermentation experiment was carried out under the experimental conditions indicated in Table 1. In total six treatments were included as follows: Control (no addition of EO/antibiotic), the unprotected BOT oil at 1.85 mg/mL and 3.70 mg/mL, the MBOT oil at 3.5 mg/mL (≈ 0.463 mg of unprotected BOT/mL) and 7.0 mg/mL (≈ 0.93 mg of unprotected BOT/mL), and colistin treatment at 2.0 μg/mL. The unprotected BOT and MBOT were tested at those concentrations because in our previous *in vitro* studies, these concentrations were the MIC and MBC for *E. coli* and did not cause the inhibition or death of *Lactobacillus* sp. (Ambrosio et al., 2019, 2020). Colistin was tested at that concentration because the MIC for this antibiotic on *E. coli* has been previously reported as ≤ 2.0 μg/mL (CLSI, 2012; EUCAST, 2019). In addition, according to JECFA (2016), the average daily intake of colistin for livestock animals was established on the basis of a colistin MIC_50_ of 1 μg/mL for *E. coli*. For this experiment, the serum bottles containing the corresponding stomach, ileum, and colon media were taken from refrigeration, warmed at 39°C and then 1 mL of each unprotected BOT, MBOT, or colistin stock solutions was added to the bottles using a syringe in order to reach the working EO/antibiotic concentrations. Bottles were shaken for homogenizing the EO/antibiotic solutions with the media. Subsequently, to start the fermentation assay, 1.5 mL of the fresh inoculum from each gut location was added to the bottles containing the respective media with the EO/antibiotic treatments using a syringe. Once bottles were fully set-up, they were stirred again for full homogenization and finally brought to incubation at 39°C for 72 h. Three replicates per media and treatment were performed in this experiment. During the incubation period, samples were taken at 0, 12, 18, 24, 48, and 72 h of fermentation.

**Table 1:** *In vitro* batch incubation conditions simulating the pig gastric, ileal, and colonic fermentation.

	**Stomach simulation**	**Ileum simulation**	**Colon simulation**
Inoculum (mL)*	1.5	1.5	1.5
Medium and headspace	Aerobic (free air)	Anaerobic (N_2_/CO_2_, 80:20 v/v)	Anaerobic (N_2_/CO_2_ 80:20 v/v)
pH	3	6	6.5–7
Temperature (°C)	39	39	39
Duration	72 h	72 h	72 h
**Treatments mg/mL**			
Control	0	0	0
BOT	1.85, 3.70	1.85, 3.70	1.85, 3.70
MBOT	3.5, 7.0	3.5, 7.0	3.5, 7.0
Colistin	0.002	0.002	0.002

^*^Once the inoculum was added to the corresponding media, an aliquot was immediately taken which represented the sample at time 0 h, referred to as “Starpoint” of the fermentation.

#### DNA extraction, PCR amplification, and sequencing

Total bacterial DNA was isolated from the pellet of 1.5 mL stomach, ileum, and colon fermentation samples. The pellets were obtained by centrifugation at 10,000 *x g* for 10 min. The DNA isolation protocol comprised the following steps: the pellet was resuspended in 650 μL Stool Transport and Recovery (STAR) buffer (Roche Diagnostics Nederland BV, Almere, the Netherlands) in a tube containing zirconia-silica beads (0,1 mm) (Sigma) and glass beads (2.5 mm) (Sigma), then the tubes were homogenized 3 times at 5.5 m/s for 60 s in a bead-beater (Berlin Technologies, CNIM, Montigny-le-Bretonneux, France). The homogenized samples were incubated at 95°C for 15 min and centrifuged at 4°C for 5 min (10,000 x g). Supernatants were taken, and the DNA was isolated using the Maxwell 16 Instrument (Promega, Leiden, the Netherlands) as described by van Gastelen et al. (2017). Amplification of the V5–6 hypervariable region of the bacterial 16S rRNA gene was performed using universal primers (784 f: 5′-[AG]GGATTAGATACCC-3′, 1064 r: 5′-CGAC[AG][AG]CCATGCA[ACGT]CACCT-3′) containing unique barcodes for each sample. Amplification reactions were successful for ileum and colon samples but not for the stomach, as the amount of DNA extracted from stomach samples was very low. Amplification by PCR was performed in triplicate, using an initial denaturation at 98°C for 30 s followed by 25 cycles of denaturation at 98°C for 10 s, annealing at 42°C for 10 s, elongation at 72°C for 10 s, and a final step of 72°C for 7 min. PCR products were analyzed by electrophoresis in a 1.3% agarose gel at 135 V to confirm the successful amplification. The replicates of the PCR products for each sample were pooled and purified using magnetic beads employing the CleanPCR kit (Clean NA, GC Biotech B.V., The Netherlands) according to the manufacturer's instructions and eluted in 35 μL of nuclease-free water (Qiagen). The DNA concentration was determined by a Qubit BR dsDNA kit using a Qubit 2.0 Fluorometer (Thermo Fisher Scientific, Waltham, MA, USA). Purified amplicons were pooled at 200 ng per sample into libraries together with positive (synthetic Mock communities of known composition) and negative controls, and the concentration of each pooled library was quantified using a Qubit 2.0 Fluorometer. Libraries were sent for sequencing using the Illumina HiSeq platform at GATC GmbH (now part of Eurofins Genomics Germany GmbH, Konstanz, Germany).

#### Quantitative real-time PCR

Quantitative real-time PCR was performed using published primer sets (Supplementary Table S2). SYBR green qPCR assays were performed with the DNA samples using an iCycler iQ real-time detection system (Bio-Rad Laboratories B.V., Veenendaal, The Netherlands). All qPCR analyses were carried out in triplicate with a reaction volume of 10 μL. The reaction mixture contained 6.25 μL of iQ SYBR green mix, 0.25 μL of each primer at 10 μM (stock concentration), 3.25 μL of PCR grade water, and 2.5 μL of the sample DNA or PCR grade water. The amplification program was comprised of an initial denaturation at 95°C for 3 min followed by 39 cycles of 95°C for 15 s, 60 or 52°C for 30 s, and 72°C for 30 s. Following amplification, melting curves were obtained by slow heating at 0.5°C/s from 65 to 95°C. Standard curves were generated with 10-fold serial dilutions of the 16S rRNA genes amplified from each targeted bacterial group: total bacteria and *E. coli*. The final copy number was calculated by multiplying raw copy numbers by the DNA dilution and the sample dilution for DNA extraction, and by dividing it by the volume of the sample (mL of culture taken for DNA extraction). Results were expressed as a logarithm of final copy numbers per mL of the culture sample (Log_10_[copies]/mL).

#### Analysis of metabolites

Short-chain fatty acid (SCFA) production was analyzed by high-performance liquid chromatography (HPLC). For this, 1.5 mL from stomach, ileum, or colon fermentation samples were taken and centrifuged at 10,000 *x* g for 10 min. Then, the supernatant was stored at −20°C until HPLC analysis. An SCFA standard curve from a stock solution (100 mM) containing lactate, formate, acetate, propionate, isobutyrate, and butyrate was prepared. Crotonate was used as the internal standard. Vials contained sample supernatants or standard curve and crotonate in a ratio 4:1 (v/v). SCFA production was measured with an LC 2030C HPLC (Shimadzu, Den Bosch, the Netherlands) equipped with a column Metacarb 67 h of 300 × 6.5 mm (Agilent, Amstelveen, the Netherlands) for the separation of organic acids. The injection volume was 20 μL. The column working temperature was 45°C, and the mobile phase was 0.01 N sulfuric acid at a flow of 1.0 mL/min. Metabolites were detected using a refractive index detector (RID-20A, Shimadzu, Den Bosch, The Netherlands).

### Bioinformatics and statistical analysis

The 16S rRNA gene sequence data were processed using NG-Tax (Ramiro-Garcia et al., 2016) version 2.0 (Poncheewin et al., 2019). Amplified sequence variants (ASVs) were defined using an open reference approach, and taxonomy was assigned to those ASVs using a SILVA 16S rRNA gene reference database version 128 (Yilmaz et al., 2014). Two different in-house mock communities (Ramiro-Garcia et al., 2016) were also sequenced as controls in order to assess the sequencing quality. The mocks were compared with their theoretical compositions by performing Pearson correlations using QIIME (Caporaso et al., 2010). The phylogenetic diversity (PD) was calculated using the Picante R package version 1.7.0 (Kembel et al., 2010) and a One-Way ANOVA test with Tukey's *post-hoc* test was performed in order to check for significant differences between treatments. Next, principal coordinate analysis (PCoA) based on weighted Unifrac distances was performed on the microbial community composition of all samples using the microbiome R package version 1.17.2 (Kembel et al., 2010). To evaluate the effect of treatments and fermentation time on the microbial community, a multivariate analysis of variance (PERMANOVA), using the Adonis test with 999 permutations was performed using vegan R package version 2.5.3 (Oksanen et al., 2018). The abundance of microbial taxa was expressed as a percentage (relative abundance) of the total 16S rRNA gene sequences. To evaluate the effect of the treatments on individual taxa, a One-way ANOVA model and a Tukey's *post-hoc* test for pairwise comparison were used to detect significant differences using the R software (version 3.6.1). The confidence level for all the analyses was 0.05.

## Results

### Total bacteria kinetics of pig gut microbial fermentation

**Figure 1:**
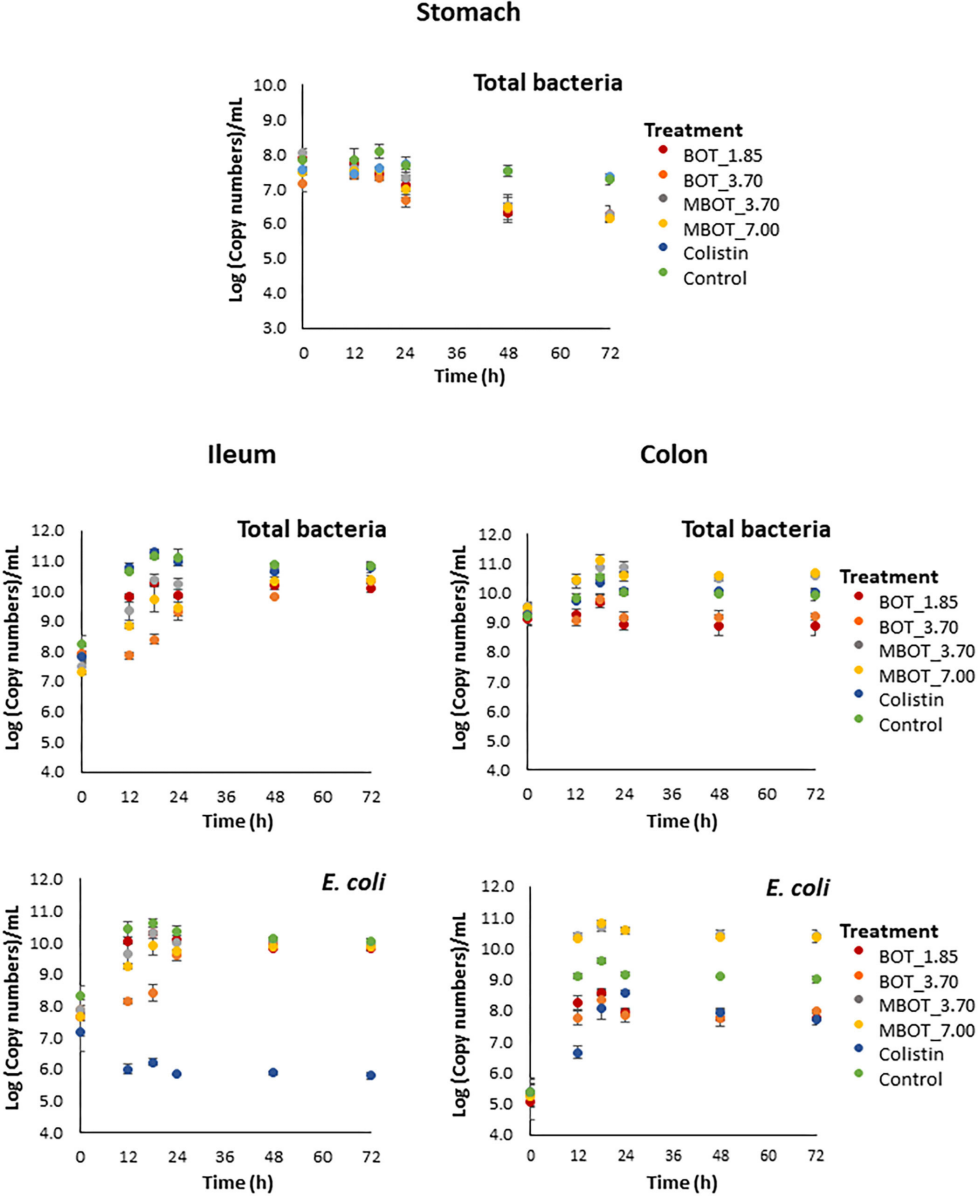
Total bacteria and *E. coli* kinetics based on the 16S rRNA gene copy numbers (Log_10_[copies]/mL) by the effect of the unprotected BOT, microencapsulated BOT (MBOT) and colistin on pig simulated stomach, ileum, and colon fermentations. Data shown are the average of triplicate incubations, with error bars indicating standard deviation.

It was observed that BOT either unprotected or microencapsulated (MBOT) altered the copy number of total bacterial 16S rRNA genes in stomach, ileum, and colon fermentations (Figure 1). In contrast, colistin did not alter total bacterial 16S rRNA gene copies for any of the three fermentation stages compared to the control. The number of total bacterial 16S rRNA gene copies in the stomach was unaltered by BOT and MBOT until 18 h after the start of the fermentation process, but both treatments reduced the number of total bacterial 16S rRNA gene copies at later time points in a similar manner, compared to the control. Regarding ileal fermentation, the unprotected BOT at 1.85 mg/mL and the MBOT (3.5 and 7.0 mg/mL) had a slight reductive effect on total bacterial 16S rRNA gene copies until 24 h of fermentation compared to the control. The higher concentration of the unprotected BOT oil (3.70 mg/mL) caused a bacteriostatic effect until 12 h of fermentation. In colon fermentation, the unprotected BOT decreased total bacterial 16S rRNA gene copies while MBOT increased it compared to the control.

### Diversity and composition of ileal and colon microbial fermentation

**Figure 2:**
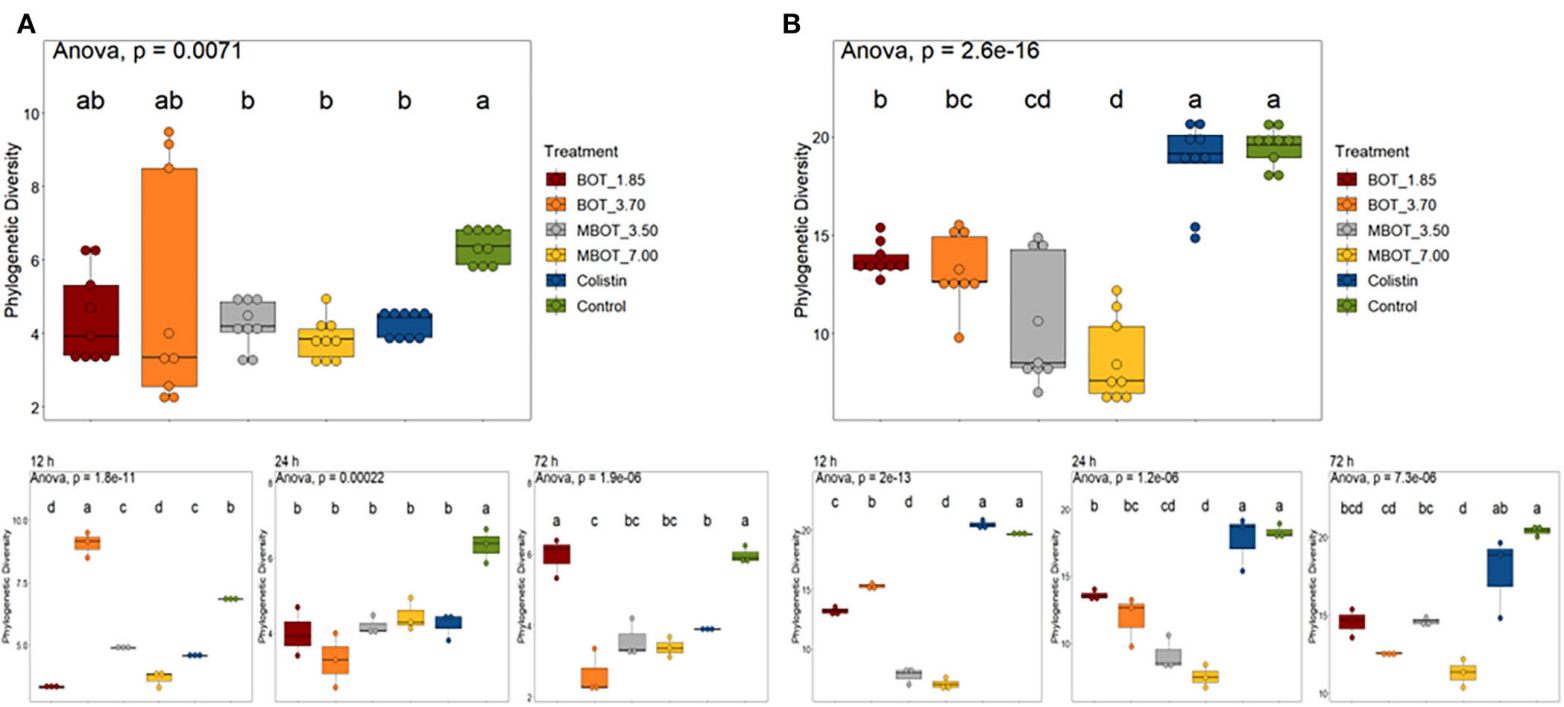
Box plot of phylogenetic diversity of **(A)** ileum and **(B)** colon microbiota during fermentations in the presence of the unprotected BOT, microencapsulated BOT (MBOT), or colistin. The different superscript letters indicate significant differences (*p* < 0.05) between any pair of groups calculated by one-way ANOVA with Tukey's *post-hoc* test.

With respect to ileal fermentations, overall, during the whole incubation, MBOT (either at 3.70 or 7.00 mg/mL) as well as colistin incubations showed significantly lower phylogenetic diversity (PD) compared to the control but were similar to those supplemented with the unprotected BOT (Figure 2A). However, the time of fermentation (12, 24, and 72 h) influenced progressive changes of PD by the effect of the unprotected BOT, MBOT, and colistin when compared to the control (Figure 2A). Regarding the colonic microbial fermentation, the unprotected BOT and the MBOT significantly reduced the PD in contrast to the control and colistin treatments (Figure 2B), but the MBOT caused a higher reduction of the PD. Colistin did not change the PD compared to the control during the whole fermentation term.

**Figure 3:**
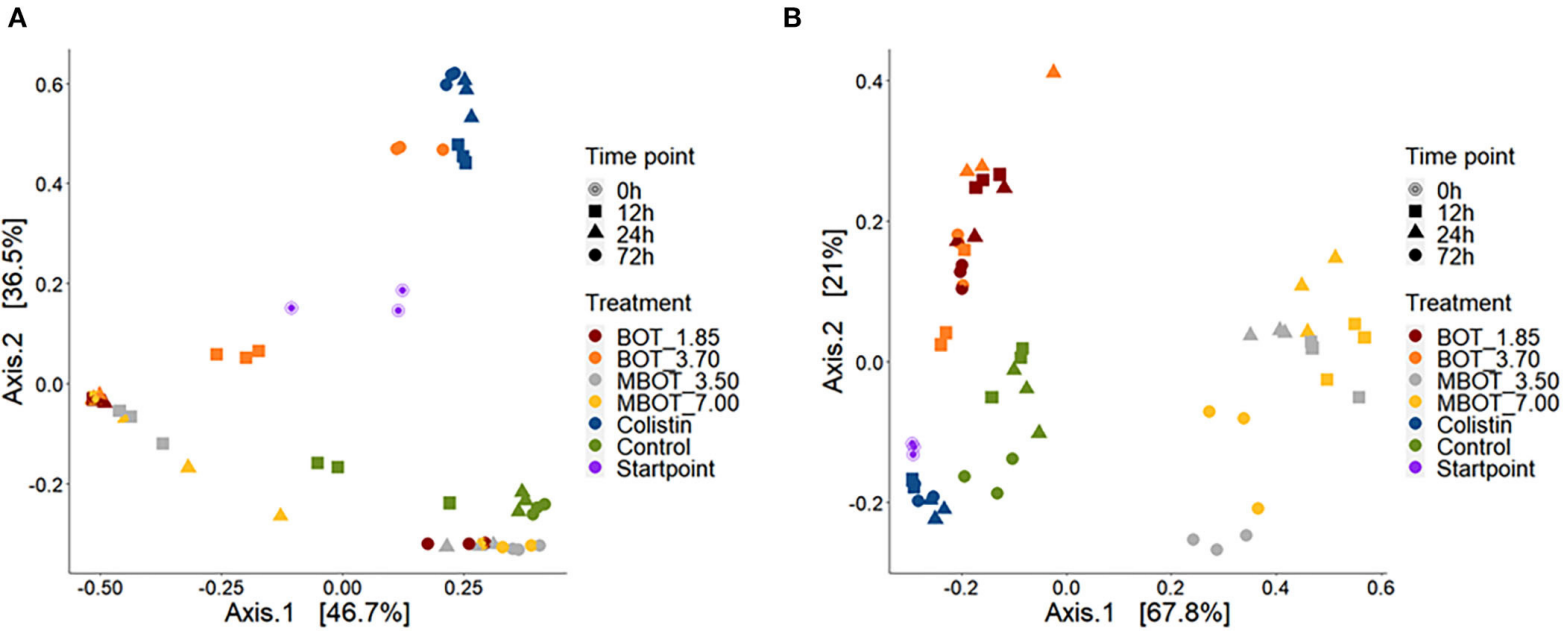
PCoA plots based on weighted UniFrac dissimilarities of the microbial composition of pig ileum **(A)** and colon **(B)** fermentations as affected by unprotected BOT, microencapsulated BOT (MBOT) and colistin treatments and the time of fermentation. “Startpoint” of fermentation refers to the composition at 0 h. Percentages at the axes indicate the amount of variation explained by the first two principal coordinates.

Subsequently, we explored the variations in ileal and colonic microbiota composition and the degree of similarity between them at the ASV level as the effect of the unprotected BOT oil, the MBOT, and colistin (Figure 3). Principal coordinate analysis (PCoA), based on weighed UniFrac distances matrices, revealed that the unprotected BOT, the MBOT, and colistin significantly affected in a different manner the bacterial composition in both, ileal and colonic fermentations, as supported by PERMANOVA and Adonis tests (Table 2). In addition, the bacterial composition was significantly altered by the time of fermentation within each treatment, since a clear clustering by incubation time was also observed in the PCoA plot for both, ileal and colonic fermentations (*p*-values Adonis test < 0.05; Figure 3). These results suggested that the bacterial community composition of both ileal and colonic microbial fermentations was significantly changed by the unprotected BOT and MBOT compared to the control and the colistin treatment.

**Table 2:** Effects of the experimental factor on *in vitro* fermentation pig microbiota.

	**Factor**	**Ileum**	**Colon**
PERMANOVA and Adonis test	Treatment	R^2^ = 0.50	R^2^ = 0.78
		p = 0.001	p = 0.001
	Time point	R^2^ = 0.25	R^2^ = 0.09
		p = 0.001	p = 0.001
	Treatment × Time point	R^2^ = 0.23	R^2^ = 0.08
		p = 0.001	p = 0.001

Adonis based on weighted UniFrac distances.

#### Taxonomical distribution

**Figure 4:**
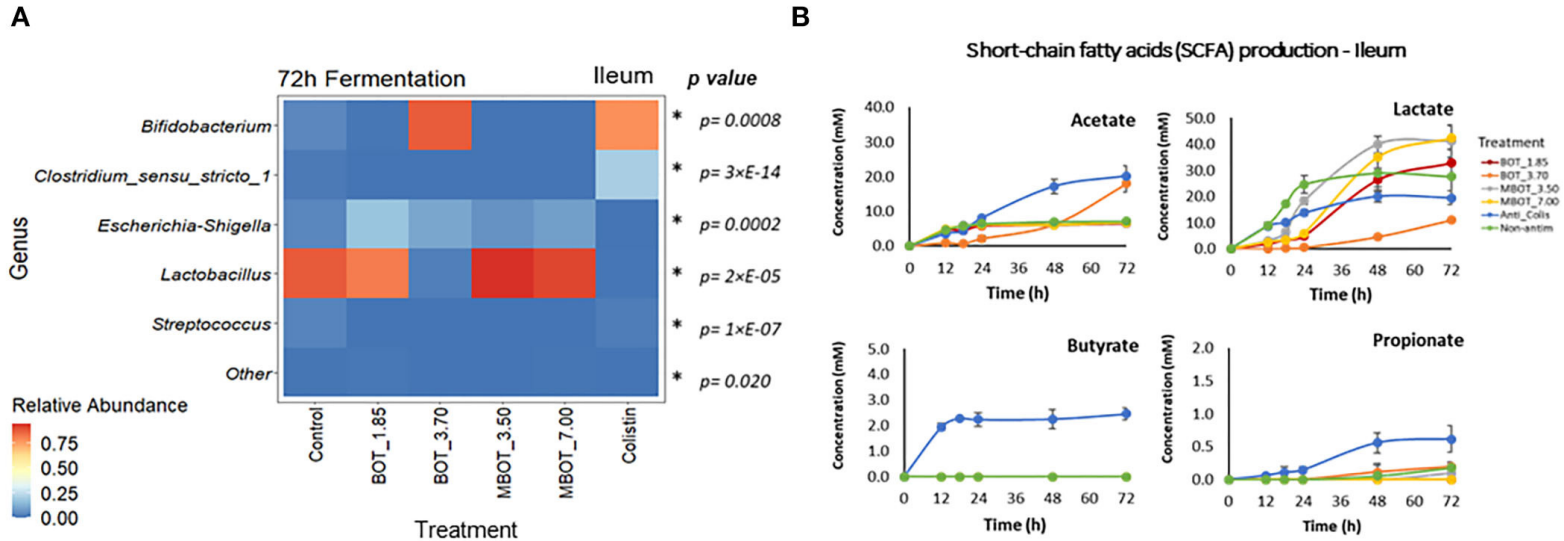
Heat map of the relative abundance of the top-5 genera present in ileum microbial fermentation **(A)** and short chain fatty acid (SCFA) production **(B)** as affected by unprotected BOT, microencapsulated BOT (MBOT), and colistin after 72 h of fermentation. *p*-values were calculated by a one-way ANOVA model and asterisks (*) indicate significant differences (*p* < 0.05). Vertical bars indicate the standard deviation.

Finally, we further explored the taxonomic distribution of specific bacterial taxa in both ileal and colonic microbial fermentations. Based on the relative abundance, we observed dynamic variations of the main phyla and genera being affected by the citrus EO treatment throughout ileal and colonic fermentations (Supplementary Figures S1, S2). For the ileal fermentation, we evaluated the changes for the top-3 phyla at the end of the fermentation (72 h), as shown in Table 3. Among these, *Firmicutes* was the most abundant phylum in the control treatment (92%), but its abundance was significantly lower in the unprotected BOT at 3.70 mg/mL and colistin treatments, while both treatments had a higher relative abundance of *Actinobacteria*. Conversely, *Actinobacteria* was not detected with MBOT. Moreover, the unprotected BOT had a higher relative abundance of *Proteobacteria* while with colistin it was not detected. Next, we evaluated the variation in the relative abundance of the top five genera in the ileal microbial fermentations for the different treatments (Figure 4A, Table 3). *Bifidobacterium* relative abundance was higher with unprotected BOT at 3.70 mg/mL (78%) and colistin (87%) compared to the control, while with both treatments, the relative abundance of *Lactobacillus* was significantly lower or not detected. With unprotected BOT and MBOT, *Clostridium sensu stricto 1* and *Streptococcus* were no longer detected while with colistin the relative abundance of *Clostridium sensu stricto 1* was higher compared to the control. The unprotected BOT compared to MBOT and the control, resulting in a slightly higher relative abundance of *Escherichia_Shigella* while with colistin this group was not detected. Specifically, results of the estimation of *E. coli* absolute abundance (Figure 1) showed that only colistin treatment reduced *E. coli* in the ileal fermentation.

**Table 3:** Relative abundance^*^ of phyla and genera after 72 h of ileal microbiota fermentation as the effect of BOT oil treatment, both unprotected (BOT) and microencapsulated (MBOT).

**Treatment**	**Control**	**BOT_1.85**	**BOT_3.70**	**MBOT_3.50**	**MBOT_7.00**	**Colistin**
**Phylum**
*Firmicutes*	0.92 ± 0.00^a^	0.82 ± 0.06^a^	0.02 ± 0.0.4^c^	0.94 ± 0.03^a^	0.91 ± 0.05^a^	0.22 ± 0.09^b^
*Proteobacteria*	0.04 ± 0.00^bc^	0.18 ± 0.06^a^	0.11 ± 0.0.4^ab^	0.06 ± 0.03^bc^	0.09 ± 0.05^abc^	0.00 ± 0.00^c^
*Actinobacteria*	0.04 ± 0.02^b^	0.00 ± 0.00^b^	0.87 ± 0.0^a^	0.00 ± 0.00^b^	0.00 ± 0.00^b^	0.78 ± 0.09^a^
Other	0.00 ± 0.00	0.00 ± 0.00	0.00 ± 0.00	0.00 ± 0.00	0.00 ± 0.00	0.00 ± 0.00
**Genus**
*Bifidobacterium*	0.04 ± 0.02^b^	0.00 ± 0.00^b^	0.87 ± 0.01^a^	0.00 ± 0.00^b^	0.00 ± 0.00^b^	0.78 ± 0.09^a^
*Clostridium sensu stricto 1*	0.01 ± 0.00^b^	0.00 ± 0.00^b^	0.00 ± 0.00^b^	0.00 ± 0.00^b^	0.00 ± 0.00^b^	0.20 ± 0.08^a^
*Escherichia_Shigella*	0.04 ± 0.01^bc^	0.17 ± 0.05^a^	0.11 ± 0.04^ab^	0.06 ± 0.03^bc^	0.09 ± 0.05^abc^	0.00 ± 0.00^c^
*Lactobacillus*	0.87 ± 0.03^ab^	0.81 ± 0.06^b^	0.02 ± 0.04^c^	0.94 ± 0.03^a^	0.91 ± 0.05^ab^	0.00 ± 0.00^c^
*Streptococcus*	0.03 ± 0.02^a^	0.00 ± 0.00^b^	0.00 ± 0.00^b^	0.00 ± 0.00^b^	0.00 ± 0.00^b^	0.02 ± 0.01^ab^
Others	0.00 ± 0.00	0.01 ± 0.00	0.00 ± 0.00	0.00 ± 0.00	0.00 ± 0.00	0.00 ± 0.00

^*^Values are means ± Standard Deviation (SD) of triplicate determinations.

The different letters indicate significant differences (p < 0.05) between any pair of groups calculated by one-way ANOVA with Tukey's *post-hoc* test.

**Figure 5:**
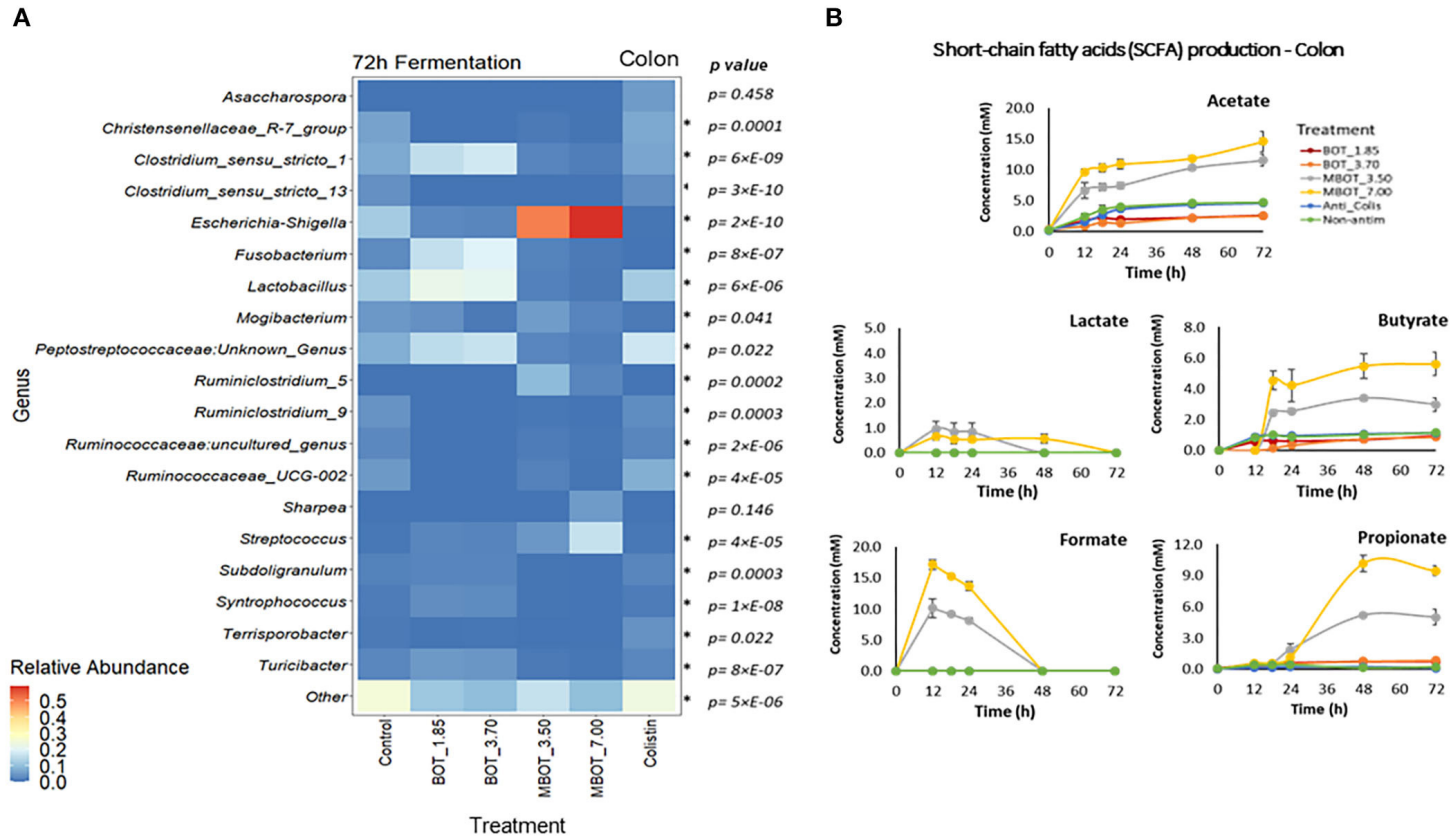
Heat map of the relative abundance of the top-19 genera present in colon microbial fermentation **(A)** and short chain fatty acid (SCFA) production **(B)** as affected by unprotected BOT, microencapsulated BOT (MBOT), and colistin after 72 h of fermentation. *p*-values were calculated by a one-way ANOVA model and asterisks (*) indicate significant differences (*p* < 0.05). Vertical bars indicate the standard deviation.

Regarding colonic microbial fermentation, we observed significant changes in the relative abundance of bacteria at phylum and genus level in incubations supplemented with BOT and MBOT at the end of the fermentation (72 h) as shown in Table 4. Firstly, at the phylum level, the variation in the relative abundance of the top-7 phyla was evaluated. Depending on the concentration tested, with the unprotected BOT and the MBOT, *Bacteroidetes, Euryarchaeota*, and *Synergistetes* were no longer detected compared to the control treatment. In addition, MBOT supplementation resulted in a significantly lower relative abundance of *Firmicutes* compared to all other treatments, while *Proteobacteria* was highest in its relative abundance. With the unprotected BOT, *Fusobacteria* relative abundance was higher in contrast to the control, but this phylum was not detected with colistin. Inversely, colistin *Firmicutes'* relative abundance was higher (95%) whereas *Proteobacteria's* relative abundance was lower when compared with the control. Furthermore, variations in the relative abundance of the top-19 genera were assessed (Figure 5A, Table 4). With the unprotected BOT and the MBOT depending on the concentration tested, *Clostridium sensu stricto 13, Terrisporobacter, Ruminiclostridium* 9, *Christensenellaceae* R7 group*, Ruminococcaceae* UCG 002, and *Ruminococcaceae* uncultured genus were no longer detected. Additionally, MBOT supplementation led to the significantly lower relative abundance of *Clostridium sensu stricto 1, Lactobacillus, Syntrophococcus, Subdoligranulum*, and *Turicibacter*, while the unprotected BOT resulted in higher or the same relative abundance of these genera when compared to colistin and the control. Inversely, MBOT resulted in a significantly higher relative abundance of *Streptococcus* and *Ruminiclostridium_*5 whereas unprotected BOT supplementation was associated with a higher abundance of *Fusobacterium* and *Peptostreptococcaceae* Unknown Genus (even though this was found not significant). Incubations in the presence of unprotected BOT had the lowest *Escherichia_Shigella* relative abundance (although not significant compared to the control), while with colistin, this group was not detected. Specifically, unprotected BOT was as efficient as colistin in decreasing *E. coli's* total abundance (Figure 1). In contrast, the MBOT supplementation resulted in a higher abundance of *Escherichia_Shigella* and *E. coli*, even at higher levels than the control. Therefore, at the genus level, it was more evident that the MBOT caused a significant reduction of several bacterial taxa than the unprotected BOT. Alternatively, or in addition, the increase in the relative abundance of some colonic bacteria by the MBOT would indicate that, besides the alterations by the delivered EO, the microcapsules (starch-modified and chitosan) could have worked as an additional carbon source to promote the growth of these bacteria.

**Table 4:** Relative abundance^*^ of phyla and genera after 72 h of colon microbiota fermentation as the effect of BOT oil treatment, both unprotected (BOT) and microencapsulated (MBOT).

**Treatment**	**Control**	**BOT_1.85**	**BOT_3.70**	**MBOT_3.50**	**MBOT_7.00**	**Colistin**
**Phylum**
*Actinobacteria*	0.01 ± 0.01^a^	0.02 ± 0.01^a^	0.02 ± 0.01^a^	0.00 ± 0.00^a^	0.00 ± 0.00^a^	0.01 ± 0.01^a^
*Bacteroidetes*	0.02 ± 0.01^ab^	0.00 ± 0.00^b^	0.00 ± 0.00^b^	0.03 ± 0.01^a^	0.00 ± 0.00^b^	0.01 ± 0.00^b^
*Euryarchaeota*	0.02 ± 0.01^a^	0.00 ± 0.00^b^	0.00 ± 0.00^b^	0.00 ± 0.00^b^	0.00 ± 0.00^b^	0.0 ± 0.00^b^
*Firmicutes*	0.78 ± 0.04^b^	0.79 ± 0.02^b^	0.76 ± 0.05^b^	0.42 ± 0.06^c^	0.39 ± 0.04^c^	0.95 ± 0.02^a^
*Fusobacteria*	0.03 ± 0.01^b^	0.16 ± 0.02^a^	0.20 ± 0.06^a^	0.02 ± 0.01^b^	0.01 ± 0.00^b^	0.00 ± 0.00^b^
*Proteobacteria*	0.13 ± 0.05^b^	0.03 ± 0.00^bc^	0.03 ± 0.01^bc^	0.53 ± 0.08^a^	0.60 ± 0.04^a^	0.01 ± 0.00^c^
*Synergistetes*	0.01 ± 0.00^ab^	0.00 ± 0.00^b^	0.00 ± 0.00^b^	0.00 ± 0.00^b^	0.00 ± 0.00^b^	0.01 ± 0.01^a^
Others	0.00 ± 0.00	0.00 ± 0.00	0.00 ± 0.00	0.00 ± 0.00	0.00 ± 0.00	0.00 ± 0.00
**Genus**
*Asaccharospora*	0.00 ± 0.00^a^	0.00 ± 0.00^a^	0.00 ± 0.00^a^	0.00 ± 0.00^a^	0.00 ± 0.00^a^	0.05 ± 0.09^a^
*Sharpea*	0.00 ± 0.00^a^	0.00 ± 0.00^a^	0.00 ± 0.00^a^	0.00 ± 0.00^a^	0.05 ± 0.06^a^	0.00 ± 0.00^a^
*Christensenellaceae* R7 group	0.06 ± 0.02^a^	0.00 ± 0.00^b^	0.00 ± 0.00^b^	0.01 ± 0.01^b^	0.00 ± 0.00^b^	0.07 ± 0.03^a^
*Clostridium sensu stricto 1*	0.07 ± 0.01^b^	0.16 ± 0.00^a^	0.18 ± 0.02^a^	0.02 ± 0.01^c^	0.01 ± 0.00^c^	0.07 ± 0.02^b^
*Clostridium sensu stricto 13*	0.04 ± 0.01^a^	0.00 ± 0.00^b^	0.00 ± 0.00^b^	0.00 ± 0.00^b^	0.00 ± 0.00^b^	0.03 ± 0.00^a^
*Escherichia_Shigella*	0.12 ± 0.05^b^	0.03 ± 0.00^bc^	0.02 ± 0.01^bc^	0.51 ± 0.07^a^	0.59 ± 0.04^a^	0.00 ± 0.00^c^
*Terrisporobacter*	0.01 ± 0.00^ab^	0.00 ± 0.00^b^	0.00 ± 0.00^b^	0.00 ± 0.00^b^	0.00 ± 0.00^b^	0.04 ± 0.03^a^
*Fusobacterium*	0.03 ± 0.01^b^	0.16 ± 0.02^a^	0.20 ± 0.06^a^	0.02 ± 0.01^b^	0.01 ± 0.00^b^	0.00 ± 0.00^b^
*Lactobacillus*	0.12 ± 0.01^b^	0.22 ± 0.03^a^	0.21 ± 0.05^a^	0.02 ± 0.00^c^	0.01 ± 0.00^c^	0.12 ± 0.05^b^
*Mogibacterium*	0.05 ± 0.01^a^	0.04 ± 0.04^a^	0.01 ± 0.00^a^	0.05 ± 0.01^a^	0.02 ± 0.00^a^	0.01 ± 0.00^a^
Others	0.25 ± 0.03	0.11 ± 0.01	0.10 ± 0.01	0.16 ± 0.01	0.10 ± 0.02	0.24 ± 0.05
*Peptostreptococcaceae* unknown genus	0.08 ± 0.01^a^	0.15 ± 0.01^a^	0.17 ± 0.02^a^	0.02 ± 0.00^a^	0.01 ± 0.00^a^	0.17 ± 0.15^a^
*Ruminiclostridium* 5	0.00 ± 0.00^b^	0.00 ± 0.00^b^	0.00 ± 0.00^b^	0.09 ± 0.03^a^	0.02 ± 0.04^a^	0.00 ± 0.00^b^
*Ruminiclostridium* 9	0.04 ± 0.00^a^	0.00 ± 0.00^b^	0.00 ± 0.00^b^	0.00 ± 0.00^b^	0.00 ± 0.00^b^	0.03 ± 0.02^a^
*Ruminococcaceae* UCG 002	0.05 ± 0.01^ab^	0.00 ± 0.00^c^	0.00 ± 0.00^c^	0.02 ± 0.01^bc^	0.00 ± 0.00^c^	0.08 ± 0.03^a^
*Ruminococcaceae* uncultured genus	0.03 ± 0.00^a^	0.00 ± 0.00^c^	0.00 ± 0.00^c^	0.01 ± 0.00^b^	0.00 ± 0.00^bc^	0.02 ± 0.01^a^
*Streptococcus*	0.004 ± 0.00^b^	0.02 ± 0.00^b^	0.02 ± 0.00^b^	0.05 ± 0.01^b^	0.16 ± 0.06^a^	0.004 ± 0.00^b^
*Subdoligranulum*	0.02 ± 0.00^a^	0.02 ± 0.01^a^	0.02 ± 0.00^a^	0.001 ± 0.00^b^	0.00 ± 0.00^b^	0.02 ± 0.01^a^
*Syntrophococcus*	0.01 ± 0.00^b^	0.03 ± 0.00^a^	0.03 ± 0.00^a^	0.002 ± 0.00^c^	0.0004 ± 0.00^c^	0.01 ± 0.01^b^
*Turicibacter*	0.02 ± 0.00^b^	0.05 ± 0.01^a^	0.05 ± 0.01^a^	0.01 ± 0.00^c^	0.00 ± 0.00^c^	0.03 ± 0.01^b^

^*^Values are means ± Standard Deviation (SD) of triplicate determinations.

The different letters indicate significant differences (p < 0.05) between any pair of groups calculated by one-way ANOVA with Tukey's *post-hoc* test.

### SCFA production

The production of fermentation end-products such as SCFAs was evaluated for the stomach, ileal, and colonic microbial fermentations. As expected, no SCFA production was detected in the stomach incubation, since at this gut stage, mainly enzymatic activity (pepsin cleaving proteins) occurs instead of microbial activity. In the ileal incubations, it was observed that acetate and lactate were the major metabolites produced (Figure 4B). Lactate production was increased by the MBOT, which could be associated with the metabolic activity of *Lactobacillus*, whose relative abundance was slightly increased, although this increase was not significant. The unprotected BOT at 3.70 mg/mL increased acetate production but decreased lactate production. Conversely, colistin stimulated acetate, butyrate, and propionate production while it decreased lactate. With respect to colonic bacterial fermentation, overall, MBOT raised the SCFAs production (acetate, butyrate, and propionate) compared to unprotected BOT, colistin and control treatments (Figure 5B), indicating that the metabolic activity of colonic bacteria was stimulated by the MBOT. This high production of SCFAs could correspond with the increased abundance and activity of some colonic bacteria such as *Streptococcus* and *Ruminiclostridium_*5 in the colonic fermentation. Thus, these results would support the possible prebiotic functions of the wall material of microcapsules for colonic bacteria since besides stimulating their growth, it would have stimulated their metabolic activity as well.

## Discussion

EOs are recognized as a potential alternative to antibiotics as a feed additive to promote growth in livestock animals. Specifically, EOs have gained interest in the pig breeding sector for their positive impacts on growth performance and pig welfare (Franz et al., 2010; Bento et al., 2013). Through a meta-analysis, it was revealed that EOs enhanced the feed conversion rate (FCR) and kept the same average daily gain (ADG) of weaning piglets compared to antibiotics (Vanrolleghem et al., 2019). This growth-promoting effect by in-feed EOs has been suggested to be strongly related to beneficial effects of EOs on the pig gut ecosystem, such as modulation of the gut microbiota (Li S. Y. et al., 2012; Xu et al., 2018). The pig gut is characterized for having a large number of microorganisms, which play important roles in pig health and wellbeing (Canibe et al., 2019). Thus, due to their antimicrobial activity, EOs can provoke changes in the pig gut microbiota. To this end, a proper application of an EO in animal feed should be assisted by encapsulation techniques, such as microencapsulation. Besides being an efficient way to deliver the EO into the pig gut, microencapsulation can also prevent or reduce the volatilization of the EO thus extending the EO shelf life in the animal feedstuff (Bento et al., 2013).

### Total bacteria kinetics

In this study, we observed that the citrus oil, BOT, both unprotected and microencapsulated, modified the pig gut microbiota in an *in vitro* gut fermentation system. Initially, we found shifts in the total number of bacteria by the effect of the unprotected BOT and MBOT as compared to colistin and the control. In a stomach simulation, neither MBOT nor unprotected BOT altered the total number of bacteria until 18 h of fermentation, but both treatments caused a decrease in the total number of bacteria afterward in a similar manner. The lack of alterations on the total number of bacteria in the stomach simulation in a period shorter than 18 h could be considered favorable since it is not intended that the EO would be released in the stomach, thus exerting an antimicrobial effect on the bacterial community of the stomach during the gastric transit of the feedstuff, which is shorter than 18 h for weaned piglets (Snoeck et al., 2004). In contrast to our results, previously, some pure EO compounds such as carvacrol and thymol (0.5 mg/mL) were reported for significantly decreasing the number of total bacteria in a pig gastric simulation after 3 h of fermentation (Michiels et al., 2009). But, in an *in vivo* study, the administration of either unprotected or microencapsulated thymol (at 0.5 or 2 mg/g) did not alter the total number of bacteria in the stomach digest of weaned piglets (Michiels et al., 2010).

In the ileal simulation, our results showed a slight bacteriostatic effect on the total bacterial load until 12 h of fermentation, only for the unprotected BOT (3.7 mg/mL), but afterward neither unprotected BOT nor MBOT altered it. Inversely, MBOT raised the number of total bacteria in the colonic microbial fermentation in comparison to the unprotected BOT. On the contrary, several commercial EO products such as microencapsulated oregano EO (10%) (Zou et al., 2016; Cheng et al., 2018), carvacrol (0.15 mg/g of feed) (Gutiérrez et al., 2010), or encapsulated thymol + cinnamaldehyde (18%) (Li P. et al., 2012; Zeng et al., 2015) were found as not being able to alter the total bacterial load in jejunum, ileum, or colon of weaned piglets.

We observed that the antibiotic treatment, colistin, did not alter the total number of bacteria for any of the three fermentation stages compared to the control. This can be explained by the specific antibacterial spectrum that antimicrobial compounds such as antibiotics have. Each antibiotic has effect on specific target bacteria, with colistin having as a main target Gram-negative bacteria such as *Escherichia coli, Enterobacter* sp., *Shigella* sp., *Klebsiella* sp., and *Pseudomonas* sp. (Rhouma et al., 2016; El-Sayed Ahmed et al., 2020). Thus, while reducing the abundance of specific bacterial groups, other groups that are not sensitive to the effect of colistin can overgrow and the total number of bacteria in the whole community (stomach, ileum, and colin fermentation) may remain stable.

### Diversity and microbial community composition

Both the MBOT and the unprotected BOT significantly affected the diversity and composition of ileal and colonic microbial fermentations. Compared to the unprotected BOT, MBOT decreased the ileal bacterial diversity, as much as colistin. In contrast, no influence of the supplementation of EO compounds such as carvacrol on ileal microbial composition was observed in piglets (Gutiérrez et al., 2010). Regarding the colonic fermentation, both the unprotected BOT and MBOT reduced the bacterial diversity, but the MBOT showed a stronger effect. Conversely, it was reported that the administration of 2% of a commercial orange EO and limonene (the major compounds in citrus EOs) increased the diversity of the cecal and colonic microbiota of mice (Wang et al., 2019). Nonetheless, supplementation with a blend of pure EO compounds (carvacrol and thymol) did not affect the colonic microbial diversity and richness of weaned piglets (Li et al., 2018). Additionally, a clear and significant effect of the unprotected BOT, the MBOT, and colistin on the bacterial community composition of pig ileal and colonic simulated fermentations was observed in our study (*p* < 0.05), where each of the treatments altered the composition in a different manner, compared to the control. In contrast, the administration of a commercial orange EO (2%) did not alter the colonic bacterial composition of mice (Wang et al., 2019). Conversely, compositional changes in the colonic microbiota of weaned piglets by thymol and carvacrol supplementation were previously reported by Li et al. (2018).

The relative abundance of certain bacterial taxa was affected by the unprotected BOT, the MBOT and colistin in ileum and colon fermentations. At the phylum level, in ileum fermentation, the MBOT caused a full elimination of *Actinobacteria*. In contrast, the unprotected BOT at 3.70 mg/mL and colistin decreased the relative abundance of *Firmicutes* while it increased that of *Actinobacteria. Proteobacteria* was increased in relative abundance by the unprotected BOT while colistin fully eliminated this phylum. Conversely to these results, dietary supplementation with an encapsulated commercial EO product (carvacrol and thymol, at 0.12 μg/mL) led to an increase of *Firmicutes* and a decrease of *Cyanobacteria* and *Proteobacteria* relative abundances in chicken ileum microbiota (Yin et al., 2017). But, the administration of carvacrol alone (0.15 mg/g) was found to have an influence by increasing *Actinobacteria* in the ileum of weaned piglets with the concomitant decrease of *Bacteroidetes* and *Clostridium* cluster XIVa (Gutiérrez et al., 2010). At the genus level, we observed that the unprotected BOT and the MBOT exerted a strong antibacterial activity on *Clostridium sensu stricto 1* and *Streptococcus* genera, which were no longer detected, in contrast to the control and colistin. In addition, *Bifidobacterium* was not detected with MBOT. These results were in-line with previous findings by Gutiérrez et al. (2010), in which carvacrol administration was found to decrease species from the genera *Streptococcus* and *Clostridium* cluster IV in the ileum of piglets. Previously, a decreasing effect on the relative abundance of *Bifidobacterium* in the ileum of weaned piglets after administration of an encapsulated EO product containing orange, oregano, anis, and chicory EOs (2%) was reported by Kroismayr et al. (2008), although this effect was not as marked as the one observed in our study. Furthermore, the MBOT did not alter *Lactobacillus* and *Escherichia-shigella* relative abundances, whereas the unprotected BOT slightly increased the relative abundances of *Escherichia-Shigella*. In contrast to the EO treatments, colistin decreased *Lactobacillus* while it caused a lack of detection of *Escherichia-shigella*, and specifically a strong reduction of *E. coli* in the ileal fermentation. No alterations of *Lactobacillus* relative abundance in the pig small intestine (proximal, distal, or ileum) after in-feed administration of several EO compounds have been previously reported (Kroismayr et al., 2008; Michiels et al., 2010). However, the supplementation with a microencapsulated oregano EO led to a decrease in the counts of *E. coli* without altering the *Lactobacillus* count in the ileum microbiota of growing-finishing pigs (Cheng et al., 2018). It has been suggested that a positive modulatory effect of the gut microbiota of weaned piglets by EO supplementation is the increase in the ratio of *Lactobacillus:Enterobacteria* in the ileum (Manzanilla et al., 2004). However, a reverse effect was observed for the unprotected BOT and MBOT in our *in vitro* trial.

Regarding colon fermentation, at the phylum level, we observed that with the unprotected BOT and the MBOT *Bacteroidetes, Euryarchaeota*, and *Synergistetes* were no longer detected. Additionally, the MBOT led to the decreased relative abundance of *Firmicutes* while the relative abundance of *Proteobacteria* was increased. Colistin increased the relative abundance of *Firmicutes*, decreased that of *Proteobacteria*, and eliminated *Fusobacteria*. Our results on the MBOT were in line with the effect at the phylum level found for limonene administration (2%), which mainly reduced *Firmicutes* relative abundance in the colon of mice (Wang et al., 2019). In contrast, supplementation with thymol + carvacrol led to an increase in *Firmicutes'* relative abundance while it reduced that of *Bacteroidetes* in the colon of weaned piglets (Li et al., 2018). The antibacterial activity of the unprotected BOT and the MBOT on the colonic bacteria was much stronger at the genus level, and overall, we observed that the MBOT exerted more remarkable effects compared to the unprotected BOT. Depending on the tested concentration, the unprotected BOT and the MBOT caused the lack of detection of six of the 19 most abundant genera compared to the control and colistin. Additionally, the MBOT caused a significant reduction in the relative abundance of *Clostridium sensu stricto 1, Lactobacillus, Syntrophococcus, Subdoligranulum*, and *Turicibacter*, while the unprotected BOT increased them and *Fusobacterium*. In addition, the MBOT tended to increase *Streptococcus* and *Ruminiclostridium_*5 abundances. Particularly, an increase in *Streptococcus* abundance in pig colon by the administration of an EO product (carvacrol + thymol) was suggested as an indicator of an improved intestinal health (Li et al., 2018). It has been indicated that the increase of other beneficial bacteria such as *Ruminococcus, Lactobacillus*, and *Megasphaera* with the simultaneous reduction of *Enterobacteriaceae* in the colon by thymol and carvacrol supplementation would be associated with low diarrhea incidence and enhanced pig performance (Li et al., 2018). Opposite to our results, the administration of an orange EO and isolated compounds normally present in this kind of EO (limonene and linalool) was found to increase beneficial bacteria such as *Lactobacillus, Parabacteroides*, and *Barnesiella* in the colon of mice, leading to a positive regulation of the intestinal microbiota (Wang et al., 2019). Furthermore, our results showed that only the unprotected BOT efficiently decreased *Escherichia_Shigella* relative abundance, while when using colistin, they were no longer detected. Both treatments also specifically diminished *E. coli* in the same manner. In contrast, the MBOT increased *Escherichia_Shigella* and *E. coli* by one level of magnitude, compared to the control. Several studies have reported that for instance supplementation with several EO compounds (thymol + carvacrol/thymol + cinnamaldehyde) effectively decreased *E. coli* in colon, rectum, or feces of weaned piglets with the simultaneous increase of *Lactobacillus* at the same level as antibiotic treatments (Li P. et al., 2012; Li S. Y. et al., 2012; Zeng et al., 2015; Xu et al., 2018). Likewise, it has been reported that a commercial product having orange, oregano, anise, and chicory EOs was effective to decrease *E. coli* and *Salmonella* and increase *Lactobacillus* and *Bacillus* in piglet feces (Ahmed et al., 2013). Similarly, oregano EO supplementation was found to promote the integrity of the intestinal barrier, probably by means of modulation of the gut microbiota related to *E. coli* reduction in jejunum, ileum, and colon of growing-finishing pigs and due to the inactivation of inflammation signaling pathways (Zou et al., 2016). The modulation of the small intestinal microbiota by feeding herbal extract or EOs could indirectly lead to improvements of the digestive capacity of the small intestine, consequently affecting growth performance (Costa et al., 2013). In addition, EOs could improve the small intestinal morphology and their antioxidative capacity, being other modes of action of EOs possibly contributing to enhance pig performance (Franz et al., 2010; Cheng et al., 2018).

### Metabolite production

The composition of the gut microbial communities and their metabolite production have an effect on the health and subsequently on the nutritional status of the host (Bento et al., 2013). The activity of the intestinal microbiota can be estimated by their fermentation end-products, such as SCFAs, which derive from the fermentation of carbohydrates and serve as a source of additional energy for the host (den Besten et al., 2013; Tungland, 2018). Besides the shifts in microbiota composition, EOs can also alter the metabolism of the intestinal microbiota, such as the production of SCFAs and other metabolites. In ileum microbial fermentation, the MBOT caused a slight stimulation of lactate production, associated probably with the activity of *Lactobacillus*. Conversely, the unprotected BOT (3.7 mg/mL) reduced lactate and increased acetate, which would be related to its effect on the abundance and metabolism of *Lactobacillus, Bifidobacterium*, and *Escherichia-Shigella*. Colistin, compared to the EO treatments, greatly stimulated SCFA production. Inversely to these results, supplementation with an EO product or avilamycin was shown to not alter SCFA production (acetate, lactate, butyrate, propionate, capric acid, and valeric acid) by the ileal microbiota of weaned piglets, although a significant increase of *Bifidobacterium* by the EO treatment was observed (Kroismayr et al., 2008). Nonetheless, microencapsulated thymol (0.5 or 2.0 mg/g of feed) was reported for changing the activity of the distal small intestinal microbiota of piglets evidenced by a decrease of acetate and lactate production without clear evidence for the alterations of coliforms, *Lactobacillus* or *Streptococcus* counts (Michiels et al., 2010). Likewise, the administration of thymol + cinnamaldehyde was found to alter the ileal microbiota metabolism of broiler chickens by reducing propionate and increasing acetate and butyrate in ileum content (Cao et al., 2010).

In colon fermentation, our results have shown that the metabolic activity of colonic bacteria was modified mainly by MBOT. Overall, MBOT greatly increased the SCFA production in the colonic fermentation. Most fermentation of feed takes place in the cecum and large intestine (den Besten et al., 2013), and SCFAs are the major fermentation end-products in those gut locations. SCFAs are mainly produced in the colon from dietary fibers, which are not digested in the small intestine (Hamer et al., 2007). Increased SCFA production in colon fermentation supplemented with MBOT could indicate that the wall material of microcapsules (modified starch and chitosan) could have worked as prebiotic, which besides having stimulated the growth of some colonic bacteria, would have stimulated their metabolic activity. The higher production of butyrate by the MBOT would be an advantageous effect, since butyrate is an important colon metabolite and energy source for epithelial cells and also influences several cellular functions affecting colonic health, such as inhibition of inflammation, reinforcement of the colonic defense barrier, and decrease in oxidative stress (Hamer et al., 2007). Acetate and propionate were also greatly increased by the MBOT. An increase in acetate would be beneficial since acetate is as important as butyrate for energy supply to colonic epithelial cells. Moreover, acetate is one of the substrates for butyrate and propionate production by colonic bacteria (den Besten et al., 2013). In addition, propionate has also potential health-promoting effects comprising anti-lipogenic, cholesterol-lowering, and anti-inflammatory activity (Hosseini et al., 2011). Acetate and propionate are mainly produced by members of the *Bacteroidetes* phylum (den Besten et al., 2013), but other phyla can also produce these SCFAs (Fernández et al., 2016). In this study, the higher acetate and propionate production could probably be associated with the metabolic activity of *Escherichia-Shigella* (*Proteobacteria*), *Streptococcus*, and *Ruminiclostridium*_5 (*Firmicutes*), whose abundances were increased by the MBOT. Stimulation of SCFA production by in-feed supplementation of an EO product to weaned piglets may be directly related to the enrichment of functions involved in carbohydrate metabolism of colonic bacteria, specifically, those related to both propanoate and butanoate metabolism (Li et al., 2018). Compared to our results, the supplementation with an encapsulated EO product constituted by a blend of carvacrol, cinnamaldehyde, and capsicum oleoresin was found to modify the SCFA production in the colon of piglets, by increasing the proportion of acetate and decreasing the proportions of butyrate and valerate (Manzanilla et al., 2004). Nevertheless, and on the contrary to our results, the administration of an orange EO led to a significant reduction of the SFCA content in the colon of mice, decreasing acetate and butyrate production (Wang et al., 2019). Similarly, a commercial EO product having a blend of orange, oregano, anis, and chicory EOs was found to decrease acetate production as much as avilamycin in the colon of weaned piglets, which was further associated with the decrease of *Bifidobacterium* and *Clostridium* counts (Kroismayr et al., 2008).

### General outlook

Our results showed that MBOT and unprotected BOT altered the microbial diversity and composition, as well as the metabolic activity of the ileal and colonic microbiota in an *in vitro* fermentation system, compared to colistin and control treatments. Remarkably, higher effects were found for the MBOT compared to the unprotected BOT, as evidenced by the reduction of bacterial taxa in ileum and colon fermentations. This would indicate that the antibacterial efficiency of the unprotected BOT was enhanced by microencapsulation since the concentrations of unprotected BOT in the microcapsules were approximately fourth times lower than the concentrations tested in the treatment with the unprotected BOT. However, the impact of the effects observed for the MBOT in this *in vitro* fermentation trial on the pig gut heath and pig performance still need to be elucidated in future *in vivo* studies. To date, the outcomes on the modulation of the pig gut microbiota by the antimicrobial effect of EOs are variable and specific depending on the evaluated EO or EO compounds. An important aspect to be pointed out as well is that most of the EO products evaluated as a potential alternative to antibiotics in the pig production sector cited in the literature and commercially available, consist of isolated single EO compounds, where thymol and carvacrol are the main compounds applied. Nevertheless, the application of an isolated EO compound instead of a whole EO could result in the selection of bacteria resistant to it in the short term. Gut bacteria could easily develop a mechanism to repel the effect of an EO compound as is the case for most antibiotics, which consist of an isolated single compound too. In fact, antibacterial resistance to carvacrol has begun to be reported (Berdejo et al., 2019). Thus, the use of a whole EO would be more recommendable since it is rich in a large number of different compounds which may inactivate bacteria by exerting different modes of action, making it more difficult for bacteria to easily develop mechanisms to resist the activity of a whole EO.

## Data availability statement

The datasets presented in this study can be found in online repositories. The names of the repository/repositories and accession number(s) can be found at: https://www.ebi.ac.uk/ena/browser/submit, PRJEB53052.

## Author contributions

CA: conception and drafting of the work, perform laboratory work, analysis, and interpretation of results, and writing and design of this manuscript. IA: expertise and support to perform microencapsulation of the EO used in this study. CW: help with laboratory analysis and critical manuscript review. RG: R training, guidance, and critical manuscript review. SA: laboratory training and critical manuscript review. CC: guidance and critical manuscript review. ED: conception and drafting of the work, guidance in all stages of the work, and critical review and final approval of the last version of this manuscript to be published. HS: conception and drafting of the work, analysis and interpretation of results, guidance in all stages of the work, critical review, and final approval of the last version of this manuscript to be published. All authors have given the final approval for the last version of this manuscript to be published.

## Conflict of interest

The authors declare that the research was conducted in the absence of any commercial or financial relationships that could be construed as a potential conflict of interest.

## Publisher's note

All claims expressed in this article are solely those of the authors and do not necessarily represent those of their affiliated organizations, or those of the publisher, the editors and the reviewers. Any product that may be evaluated in this article, or claim that may be made by its manufacturer, is not guaranteed or endorsed by the publisher.

## Supplementary material

Supplementary Figure S1 Changes in the taxonomical distribution at phylum level of ileum **(A)** and colon **(B)** microbial fermentations by the effect of the unprotected BOT, microencapsulated BOT (MBOT), and colistin. “Startpoint” of fermentation refers to the taxonomical distribution of bacteria at 0 h.

Supplementary Figure S2 Changes in the taxonomical distribution at genus level of ileum **(A)** and colon **(B)** microbial fermentations by the effect of the unprotected BOT, microencapsulated BOT (MBOT), and colistin. “Startpoint” of fermentation refers to the taxonomical distribution of bacteria at 0 h.

Supplementary Table S1 Composition of media used for simulating *in vitro* stomach, ileal, and colonic conditions.

Supplementary Table S2 Primers used for real-time PCR.
