# Supplementary figures and images for: Exploring the effect of a microencapsulated citrus essential oil on in vitro fermentation kinetics of pig gut microbiota
Source: Front Microbiol. 2022 Aug 29;13:952706. doi: 10.3389/fmicb.2022.952706 (PMC9465239; doi:10.3389/fmicb.2022.952706)

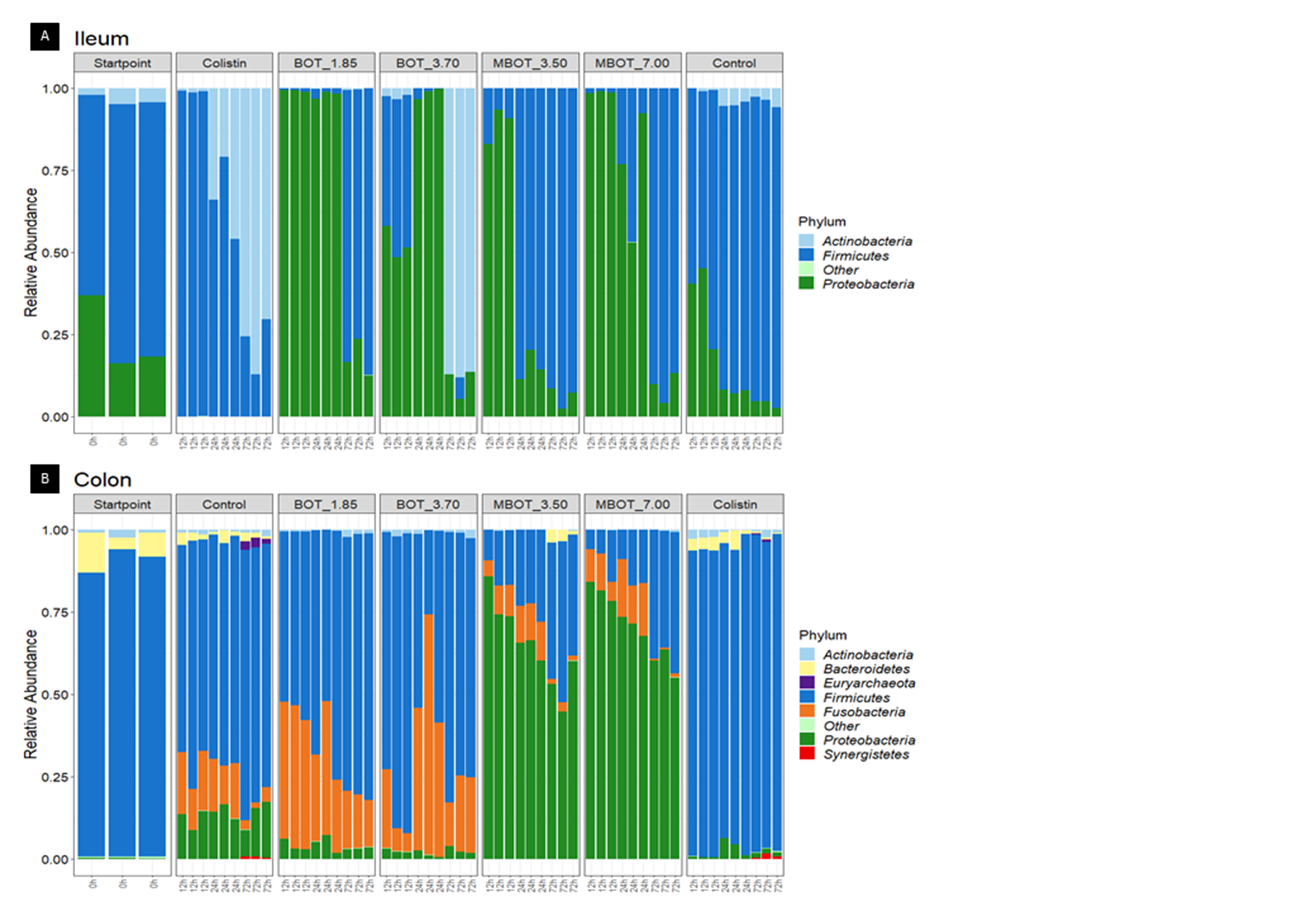

Supplement: Supplementary Figure S1 — Changes in the taxonomical distribution at phylum level of ileum (A) and colon (B) microbial fermentations by the effect of the unprotected BOT, microencapsulated BOT (MBOT), and colistin. “Startpoint” of fermentation refers to the taxonomical distribution of bacteria at 0 h. [file Image_1.tif]

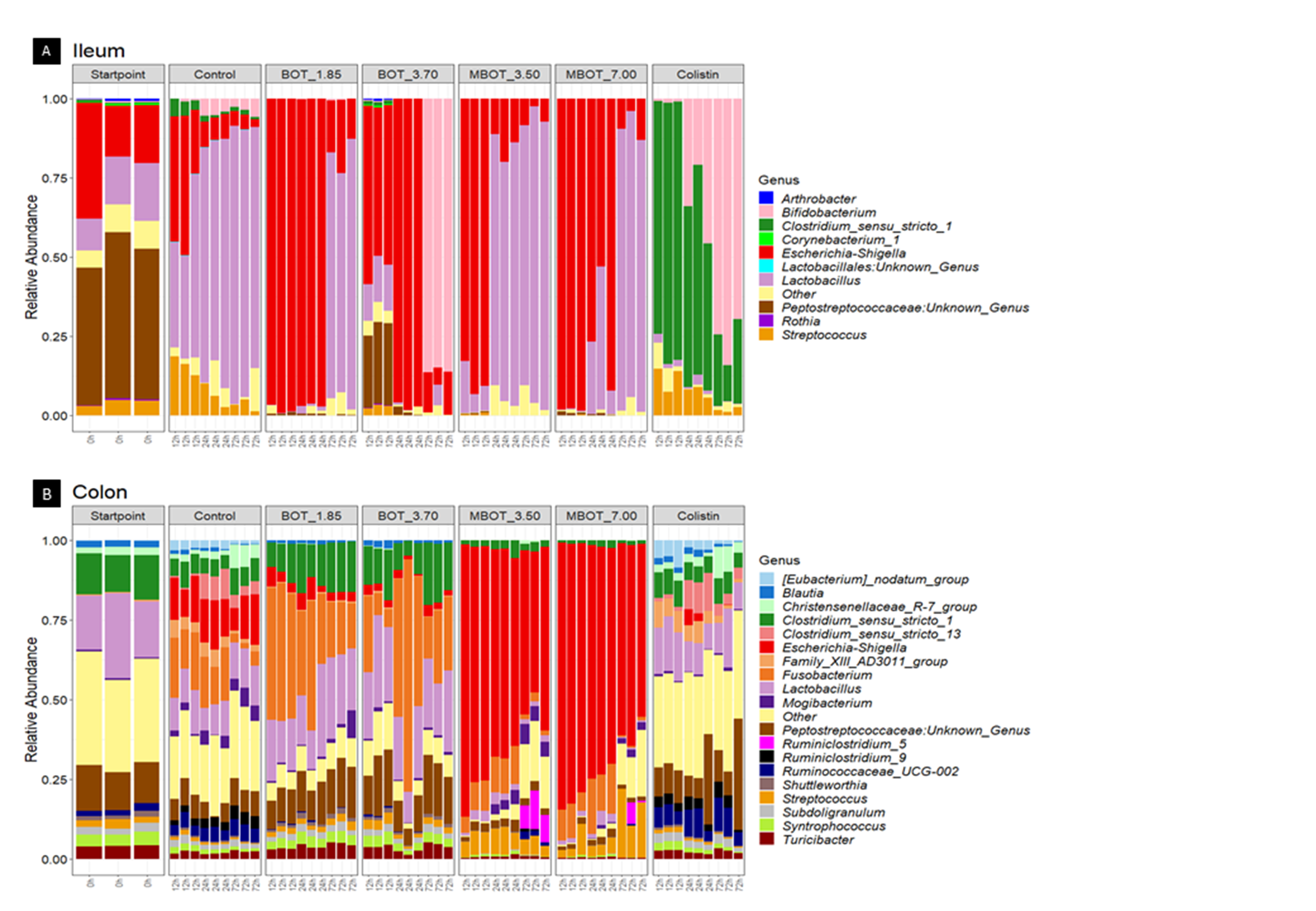

Supplement: Supplementary Figure S2 — Changes in the taxonomical distribution at genus level of ileum (A) and colon (B) microbial fermentations by the effect of the unprotected BOT, microencapsulated BOT (MBOT), and colistin. “Startpoint” of fermentation refers to the taxonomical distribution of bacteria at 0 h. [file Image_2.tif]
