# Supplementary material for: Exploring the effect of a microencapsulated citrus essential oil on in vitro fermentation kinetics of pig gut microbiota
Source: Front Microbiol. 2022 Aug 29;13:952706. doi: 10.3389/fmicb.2022.952706 (PMC9465239; doi:10.3389/fmicb.2022.952706)
Supplement: Supplementary Table S1 — Composition of media used for simulating in vitro stomach, ileal, and colonic conditions. [file Table_1.DOCX]

**SUPPLEMENTARY TABLES**

Table S1. Composition of media used for simulating *in vitro* stomach, ileal and colonic conditions

| Pig gut stage simulation | Ingredients/quantity | Pre-flushing | Sterilization |
| --- | --- | --- | --- |
| Stomach | *Basal solution* (for 1L of distilled water)  Proteose-peptone 8.3 g, D-glucose 3.5 g, NaCI 2.05 g, KH_2_PO_4_ 0.6 g, CaCI_2_ 0.11 g, KCI 0.37 g.  1 mL of L-cystein HCl solution (0.5g/L)  *Enzyme solution* (for 1L of distilled water)  Porcine bile 0.05 g, lysozyme 0.1 g, pepsin 13.3 mg | After boiling, to cool flushing N_2_/CO_2_***  N_2_/CO_2_ | Autoclaving  Filtering (0.2 µm) |
| Ileum | *Basal solution* (for 1L of distilled water)  Xylan 0.6 g, pectin 0.6 g, amylopectin 0.6 g, starch 5.0 g, casein 3.0 g, pancreatic digest of peptone from casein 3.0 g, K_2_HPO_4_ 2.0 g, NaHCO_3_ 0.2 g, NaCl 4.5g, MgSO_4_·7H_2_O 0.5g, CaCl_2_·2H_2_O 0.45 g, FeSO_4_·7H_2_O 0.005 g, haemin 0.01 g, bile salts 0.05 g, antifoam A 0.5 mL, tween 80 2.0 mL.  1mL of resazurin solution*  1 mL of L-cystein HCl solution (0.5g/L)  *Vitamin* (for 1L of distilled water)  Menadione 1.0 mg, biotin 2.0 mg, pantothenate 10mg, nicotinamide 5 mg, vitamin B12 0.5 mg, thiamine 4.0 mg and paraminobenzoic acid 5.0 mg.    *Trace mineral solution* (for 1L of distilled water)  EDTA 500 mg, FeSO_4_·7H_2_O 200 mg, ZnSO_4_·7H_2_O 10 mg, MnCl_2_·4H_2_O 3.0 mg, H_3_BO_3_ 30 mg, CoCl_2_·6H_2_O 20 mg, CuCl_2_·2H_2_O 1.0 mg, NiCl_2_·6H_2_O 2.0 mg, NaMoO_4_·2H_2_O 3.0 mg. | After boiling, to cool flushing N_2_/CO_2_  N_2_/CO_2_  N_2_/CO_2_ | Autoclaving  Filtering (0.2 µm)  Filtering (0.2 µm) |
| Colon | *Basal solution* (per 1L of distilled water)  KCl 0.6 g, NaCl 0.6 g, CaCl_2_·2H_2_O 0.2 g, MgSO_4_·7H_2_O 0.5 g, pipes buffer 1.5 g, NH_4_Cl 0.54 g, trypticase 1.0 g, D-glucose 2.6 g, trace mineral solution 10 mL, Haemin** Solution 10 mL, and fatty acid solution 10 mL.  1mL of resazurin solution (0.2 g resazurin per 200 mL distilled water)  *Trace Mineral Solution* (per 1L of 0.02M HCl): MnCl_2_·4H_2_O 0.025 g, FeSO_4_·7H_2_O 0.020 g, ZnCl_2_ 0.025 g, CuCl·2H_2_O 0.025 g, CoCl_2_·6H_2_O 0.050 g, SeO_2_ 0.050 g, NiCl_2_·6H_2_O 0.250 g, Na_2_MoO_4_·2H_2_O 0.250 g, NaVO_3_ 0.0314 g and H_3_BO_3_ 0.250 g.  *Fatty Acid Solution* (per 1L of 0.2M NaOH): Acetic acid 6.85 mL, propionic 3.0 mL, butyric 1.84 mL, *iso*-butyric 0.47 mL, 2-methyl-butyric 0.55 mL, valeric 0.55 mL and *iso*-valeric acid 0.55 mL.  *The reducing agent* (1L of boiled distilled water)  Na2S·9H2O 20.5 g, cysteine HCl 20.5g. Prepared into a fume cupboard due to the inhalation of toxic fumes.  *The bicarbonate solution* (1L of boiled distilled)  Na2CO3 (sodium carbonate anhydrous) 82 g  *The vitamin/phosphate solution* (per 1L of KH_2_PO_4_ solution (54.7 g/L))  Biotin 0.0204 g, folic acid 0.0205 g, calcium dpantothenate 0.1640 g, nicotinamide 0.1640 g, riboflavin 0.1640 g, thiamin HCl 0.1640 g, pyridoxine HCl 0.1640 g, *para*-amino benzoic acid 0.0204 g, cyanocobalamin (vitamin B12) 0.0205 g. | After boiling, to cool flushing N_2_/CO_2_  N_2_/CO_2_  N_2_/CO_2_  N_2_  Before/after autoclaving, and prior use flush with CO_2_  N_2_/CO_2_ | Autoclaving  Filtering (0.2 µm)  Filtering (0.2 µm)  Autoclaving  Autoclaving  Filtering (0.2 µm) |

* Prepared according to (Blake et al., 2003): resazurin solution = 0.2 g resazurin per 200 mL distilled water.

** Prepared according to (Williams et al., 2005): Haemin solution = 0.1 g Haemin dissolved in small amount. 0.05M NaOH and made up to 1L of boiled distilled water with CO_2_ flushing through it.

*** N_2_ + CO_2_ at the ratio 80:20 v/v

Table S2. Primers used for real-time PCR

| Primer | Orientation | Primer sequence (5’ to 3’) | Annealing  temperature (°C) | Reference |
| --- | --- | --- | --- | --- |
| Total bacteria | Forward | GTGSTGCAYGGYYGTCGTCA | 52 | (Heinritz et al., 2018) |
|  | Reverse | ACGTCRTCCMCNCCTTCCTC |  |  |
| *Escherichia coli* | Forward | CATGCCGCGTGTATGAAGAA | 60 | (He et al., 2017) |
|  | Reverse | CGGGTAACGTCAATGAGCAAA |  |  |
